# Pink illusions and white shifts

**DOI:** 10.1177/20416695241291303

**Published:** 2024-11-25

**Authors:** Stuart Anstis, Sae Kaneko, Patrick Cavanagh

**Affiliations:** Department of Psychology, University of California San Diego, La Jolla, CA, USA; Department of Psychology, Graduate School of Humanities and Human Sciences, 12810 Hokkaido University, Sapporo, Hokkaido, Japan; Department of Psychology, Glendon College, North York, ON, Canada; CVR, York University, North York, ON, Canada; Psychological and Brain Sciences, Dartmouth College, Hanover, NH, USA

**Keywords:** color vision, afterimage, white shift

## Abstract

A rotating stimulus of alternating red and white sectors generates a faint pink fill throughout the image. The trailing cyan after images of the red sectors quickly become the brightest regions in the image, providing an index of the overall illumination that triggers a shift of the white point. Actual white areas then shift in the opposite direction and appear pink.

Anstis (2022) introduced a “pink illusion” in which a rotating red and white sectored disk induced a faint hue into a central white target. This induced color, instead of being green, was unexpectedly pink. Movie 1 shows this illusion in four versions with red, green, blue, and black sectored disks. During fixation of each disk in turn, the central white disk and ring are soon filled with a faint color that matches that disk's colored sectors when they are red or green but light gray when the sectors are blue or black. The faint fill in the center and in the ring may be also seen outside the rotating disk. Anstis (2022), using just red sectored disks, proposed that the moving sectors created a desaturated cyan afterimage in the trailing white sectors and this faint cyan afterimage then induced the opposite color, pink, via color contrast into the central disk and ring. Here, we offer a new interpretation—the colored fill results not from color induction but from a shift in the neutral white toward the color of the afterimage. We show that this depends on creating regions that appear brighter than the rest of the scene that then anchor the white point (Gilchrist et al., 1999). In our first two demonstrations, it is the afterimages that trail the rotating, colored sectors that become the brightest areas in the scene. These provide a new anchor for the white point shifting it toward the chromaticity of the afterimage.

**Movie 1A.** Fixate each central plus sign of these four movies in turn allowing 10 s or more for the afterimage to build up before moving on to the next. For the red and green disks (A and B), faint colours may be seen filling the central white circular area and ring, and the surround as well. The effect of the blue disk (C) is more variable. For the black disk (D), the central white spot and ring may fill with a light grey.

**Movie 1B.** Green Disk

**Movie 1C.** Blue Disk

**Movie 1D.** Black Disk

There are two arguments in favor of this white shift hypothesis. First, the afterimages in the white sectors do appear to be the brightest region of the image. Specifically, the response to steady white areas (the surround, the ring, and the center) is attenuated, as they are continually adapted to white. Regions with alternating colored and white sectors are only exposed to white for half the time and therefore give a stronger response when white is present. In the case of the black sectored disk, these white sectors eventually appear to be whiter and brighter than the steady white areas. Since areas that are the brightest in the field can be taken as the anchor for white (Gilchrist et al., 1999), the steady white regions are then seen as light gray. The afterimages for the other red and green colored disks are also the brightest regions in their frames and so may also serve as a white anchor, but now shifting the chromaticity of the white point toward that of the afterimage, causing the steady white areas to shift away from the afterimage, toward the chromaticity of the colored sectors. This may seem counterintuitive because there is plenty of real white throughout the much larger area of the background of the figure. Nevertheless, in this case, the brightness of the afterimages wins out in anchoring the white point. Note that we are basing our white shift proposal on the increased brightness of the afterimages whereas the anchoring principle described by Gilchrist et al. (1999) is based on areas that have the highest luminance. Brightness and luminance are tightly coupled but can differ for saturated colors, especially red and blue (Ayama & Ikeda, 1998; Shioiri & Cavanagh, 1992). In the case of the afterimages, however, we cannot make any appeal to luminance as it does not vary from the background to the afterimage areas, only brightness does. As a result, we feel that brightness is a necessary proxy for luminance when dealing with afterimages.

There is one possible exception to this white shift prediction in the three colored examples presented in Movie 1. For the disk with the blue sectors, some observers may not see the expected faint blue fill but rather an achromatic or slate gray fill. This absence of notable color to the shift may be due to the strong bias to label blue chromaticities as white (Winkler et al., 2015).

The second argument in favor of the white shift hypothesis is the spatial extent of the colored fill in the background. Note that the illusory fill is not limited to the small central disk and ring but also spreads outside the disk, suggesting again that the basic neutral white has shifted toward the afterimage color, changing the interpretation of the entire figure. The spread to the surround covers a far greater spatial extent than has been reported for the lateral inhibition that underlies color induction and color contrast (McCann & Rizzi, 2003).

Movie 2 presents a simplified version of the rotating sectored disk consisting of a single dashed ring, here only for a red ring. When fixating the center of the ring, the center area and then the whole surround may gradually fill with a slightly pink shade. The gaps in the ring may have a slight cyan tint or appear simply white. The induced pink cannot easily be attributed to color induction from these afterimages because the gaps where they are present have the same spatial size as the red ring that they interrupt. It would be highly unlikely that these small areas of desaturated cyan would have a stronger influence on the central region than the saturated red parts of the ring. Instead, the afterimages in the gaps again appear brighter than the white background (which has been adapted continuously to white) and possibly even whiter than the background. This brightest point may anchor “white” (Gilchrist et al., 1999) or at least shift it towards its chromaticity. Since the surround has less cyan than the afterimage, it will look pink.

**Movie 2A.** A. rotation. Fixate the central dot and allow 10 s or more for the afterimage to build up. There may be a faint pink colour filling the center of the ring and the surround outside the ring. B. Counterphase. This produces a similar afterimage and colour fill showing that the effect is due to the afterimage, not the motion.

**Movie 2B.** RedFlashes

We next explore whether the presence of an afterimage is crucial or is simply a means to creating regions that are reliably brighter than others in the field. In Figure 1, a desaturated, broken, cyan ring lies on an achromatic light gray background. The cyan sectors of the ring are slightly brighter than the surrounding and the brightest areas in the field. The color interpretation can be variable depending on monitors, individual viewers, and recent viewing history. For some, the cyan sectors may appear to be white and the light-gray surround may look pinkish. The variability of this percept suggests that some properties of the afterimage may make it more effective at shifting the neutral white; for example, the afterimage brightness lies outside the range that can be produced on the monitor with static presentation. These potential shifts of the white point on static images are among the many issues faced when calibrating displays to achieve the intended white balance.

**Figure 1.**
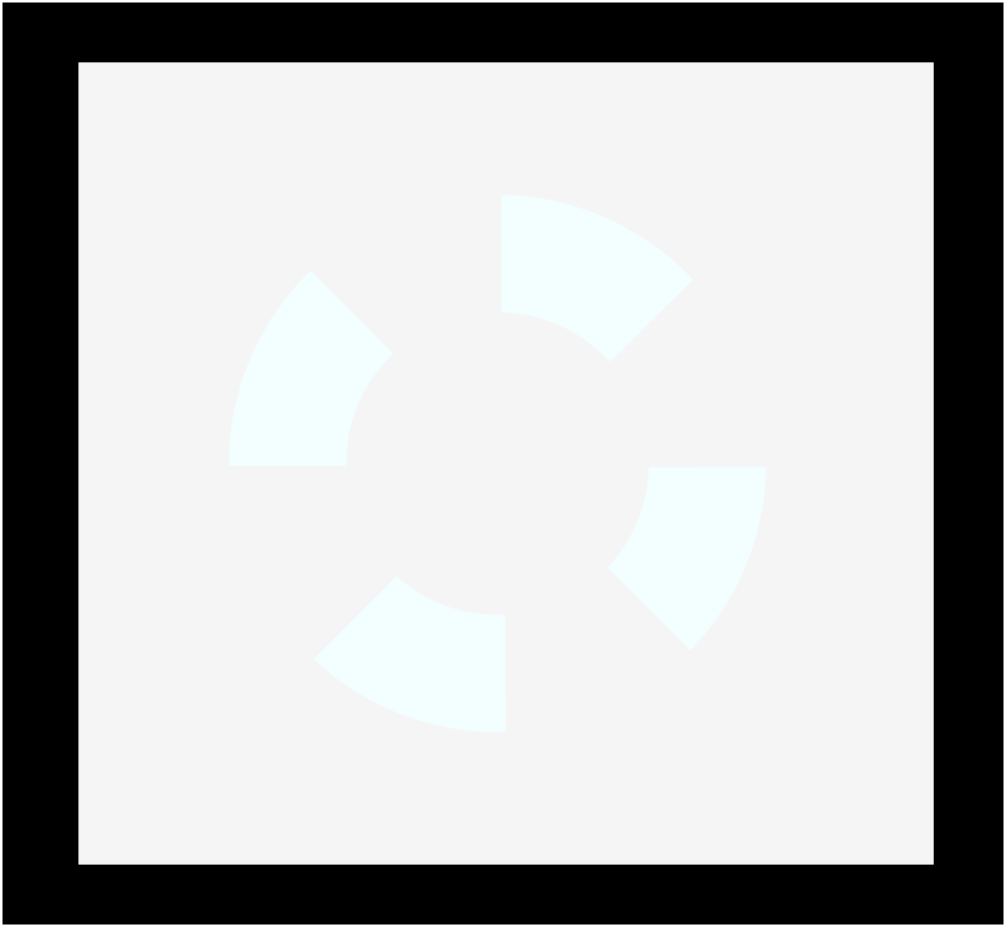
The background is a neutral, light gray while the broken ring is a slightly brighter desaturated cyan. Depending on the monitor, the gray background may appear pinkish and the cyan may appear white. On some monitors, the faint cyan regions may not be visible. If this is the case, please adjust the contrast or brightness controls until they are.

We conclude that the pink illusion from Anstis (2022) is not a case of local interactions between colors but is instead a global recalibration of the achromatic point—a “white shift.” The bright, slightly cyan patches could arise in two ways. In our case here the adaptation to the red disk or ring has desensitized red-sensitive cones, shifting the response to the white areas toward cyan at the adapted locations. Alternatively, in other stimuli, the illuminant may be slightly cyan. In either case, if the brightest region of the image provides an index of the overall illumination, the visual system may then discount it, shifting the white point toward the chromaticity of the bright region (Helmholtz, 2013). This white shift is a form of automatic chromatic gain control where the gain of the red channel, in this case, could be increased to bring the brightest region back to neutral white, thus providing for self-calibrating color vision. This would make the white surround look pink.

## References

[bibr1-20416695241291303] Anstis S. (2022). A pink illusion. Journal of Illusion, 3, 8786. 10.47691/joi.v3.8786

[bibr2-20416695241291303] Ayama M. Ikeda M. (1998). Brightness-to-luminance ratio of colored light in the entire chromaticity diagram. Color Research & Application, 23(5), 274–287. 10.1002/(SICI)1520-6378(199810)23:5<274::AID-COL4>3.0.CO;2-T

[bibr3-20416695241291303] Gilchrist A. Kossyfidis C. Bonato F. Agostini T. Cataliotti J. Li X. Economou E. (1999). An anchoring theory of lightness perception. Psychological Review, 106(4), 795–834. 10.1037/0033-295X.106.4.795 10560329

[bibr4-20416695241291303] McCann JJ. Rizzi A . (2003, January). The spatial properties of contrast. In *Color and Imaging Conference* (Vol. 11, pp. 51-58). Society of Imaging Science and Technology.

[bibr5-20416695241291303] Shioiri S. Cavanagh P. (1992). Achromatic form perception is based on luminance, not brightness. Journal of the Optical Society of America A, 9(10), 1672–1681. 10.1364/JOSAA.9.001672 1403241

[bibr6-20416695241291303] Von Helmholtz H. (2013). Treatise on Physiological Optics, Volume III (Vol. 3). Courier Corporation.

[bibr7-20416695241291303] Winkler A. D. Spillmann L. Werner J. S. Webster M. A. (2015). Asymmetries in blue–yellow color perception and in the color of ‘the dress’. Current Biology, 25(13), R547–R548. 10.1016/j.cub.2015.05.004 PMC4489998 25981792

